# Polypharmacy and Nutraceuticals in Veterans: Pros and Cons

**DOI:** 10.3389/fphar.2019.00994

**Published:** 2019-09-10

**Authors:** Tommaso Sciarra, Mario Ciccotti, Paola Aiello, Paola Minosi, Diego Munzi, Cosimo Buccolieri, Ilaria Peluso, Maura Palmery, Florigio Lista

**Affiliations:** ^1^Joint Veteran Center, Scientific Department, Army Medical Center, Rome, Italy; ^2^Department of Physiology and Pharmacology “V. Erspamer,” La Sapienza University of Rome, Rome, Italy; ^3^Research Centre for Food and Nutrition, Council for Agricultural Research and Economics (CREA-AN), Rome, Italy; ^4^National Center for Drug Research and Evaluation, Istituto Superiore di Sanità, Rome, Italy; ^5^Scientific Department, Army Medical Center, Rome, Italy

**Keywords:** veterans, polypharmacy, nutraceuticals, food–drug interactions, interprofessional interventions

The presence of multiple chronic conditions (multi-morbidity) is common in veterans, in particular among the elderly (Golchin et al., 2015). Many of veterans’ injuries have been described as a poly-trauma clinical triad, which refers to the co-occurrence (**Figure 1**) of post-traumatic stress disorder (PTSD), chronic pain, and traumatic brain injury (TBI). While the concomitant injuries (**Figure 1**) accompanying TBI may be manifold, including fractures, amputations, burns, spinal cord injury, eye injury, and auditory trauma, the two most prevalent and functionally disabling conditions may be PTSD and chronic pain (Lew et al., 2009). Although it has been recently suggested that treatment with opioids is not superior to treatment with nonopioid medications, including acetaminophen, for improving pain-related function in patients with chronic pain (Krebs et al., 2018), paracetamol pharmacokinetic is affected by nutraceuticals and some plant foods (**Figure 1**) (Abdel-daim et al., 2018).

**Figure 1:**
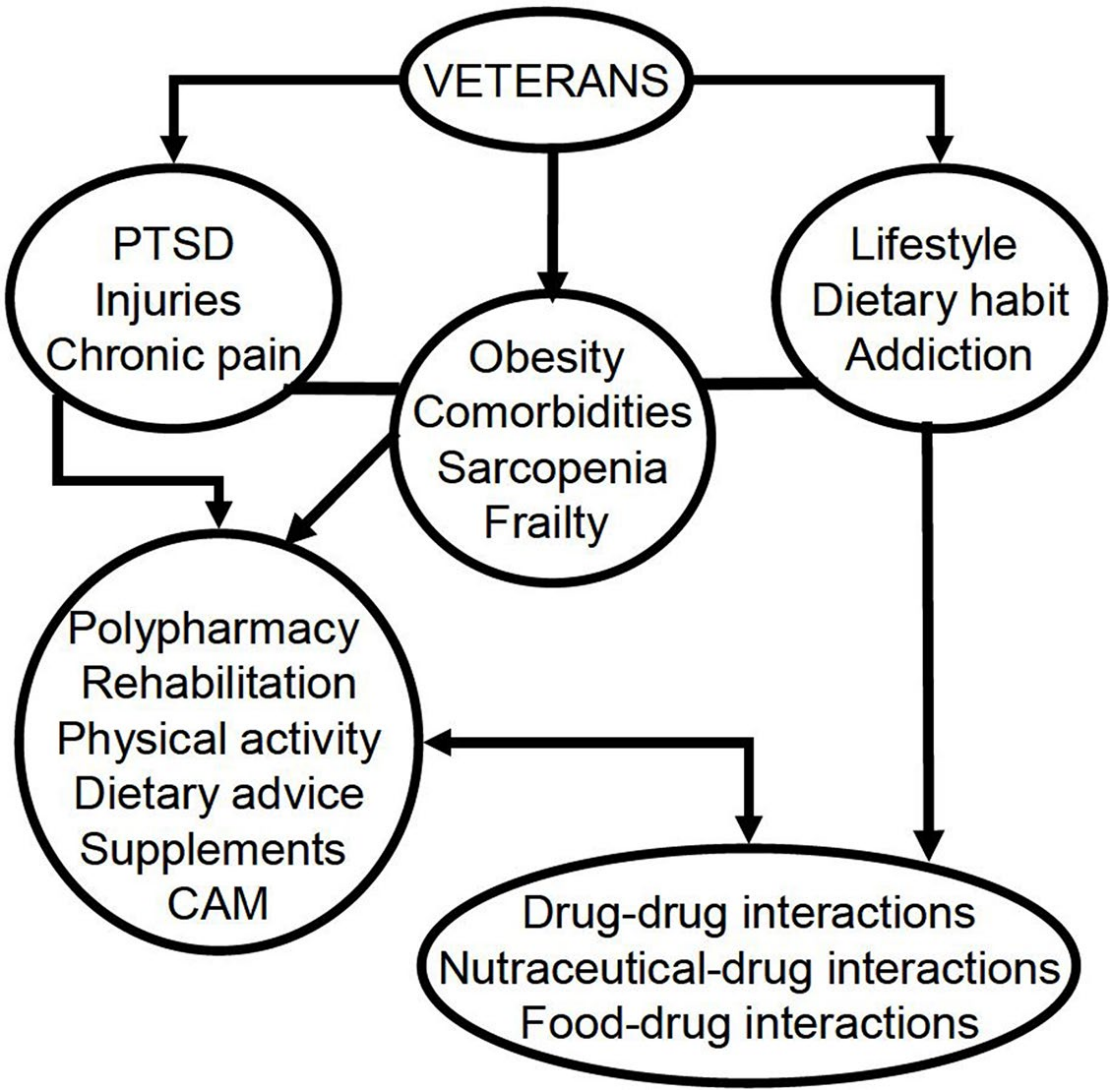
Factors that account for the need of interprofessional approaches (including physical medicine and rehabilitation clinicians, pharmacologists, and nutritionists). PTSD, post-traumatic stress disorder; CAM, complementary and alternative medicine.

Moreover, chronic pain symptoms are often comorbid with psychiatric conditions, such as depression (Runnals et al., 2013), substance use disorders (**Figure 1**) (Caldeiro et al., 2008), functional disability, and growing epidemic of prescription opioid abuse (Wilder et al., 2016). PTSD has been associated not only with cardiovascular diseases, such as hypertension (Abouzeid et al., 2012), but also with cancer (Boscarino, 2008), type 2 diabetes (Boyko et al., 2010), and poor health, including obesity (**Figure 1**) (Smith et al., 2015).

As a consequence, the use of five or more medications (polypharmacy) (**Figure 1**) to control symptoms, in order to prevent both disease complications and the development of new medical conditions, is very common in veterans, so the accumulation of multiple medications represents a critical patient safety issue. In fact, the greater the number of total prescribed medications, the greater the likelihood of prescribing a potentially harmful drug. A suitable polypharmacy can extend life expectancy and maintain quality of life when medicines are prescribed according to the best evidence and their usage is optimized. However, it has been documented that too often polypharmacy can be a detriment in case of inappropriate prescriptions (Soerensen et al., 2016) and potential prescription omissions (Rongen et al., 2016). One possible solution is deprescribing, namely, the intentional, proactive, rational discontinuation of a medication that is no longer indicated or for which the potential risk outweighs the potential benefits. The issue becomes more complicated when certain medical guidelines [e.g., those for chronic heart failure (Yancy et al., 2017)] require treatment with multiple medications to achieve the optimal clinical effect.

Furthermore, polypharmacy is sometimes associated with poor clinical outcomes, especially in older adults, including falls, frailty (**Figure 1**), impaired cognition, increased hospital admissions, and adverse drug reactions (Gnjidic et al., 2012). The most worrisome consequence of polypharmacy is the occurrence of therapeutic failures, adverse drug withdrawal events, and drug–drug interactions leading to hospitalization. All of these events are associated with similarly negative economic outcomes, such as increased drug cost and costs associated with more frequent usage of health services (Fried et al., 2014). On the other hand, the impact of some drugs on dietary habit and nutritional status is well documented (Lappin et al., 2018; Little, 2018). It is well known that polypharmacy, malnutrition, and sarcopenia are major causes of frailty (**Figure 1**) and that rehabilitation, nutrition, and interventions with mixed outcomes are important to improve disability (Singh et al., 2012; Little, 2018; Roberts et al., 2018; Wakabayashi, 2018). Despite nutritional supplements being taken into consideration in malnourishment in polypharmacy (Gaddey and Holder, 2014), another phenomenon that should not be underestimated is the trend to use vitamins and nutritional supplements instead of prescription medications. It can be assumed that costs, treatment beliefs, and/or health system distrust are the leading factors which have been influencing this trend. In the United States, especially among veterans, there is a penchant for the use of vitamins and supplements, which represent the most common form of complementary and alternative medicine (CAM) currently in use (Goldstein et al., 2014).

According to a report on the website (US Food and Drug Administration, 2008), many patients use them in addition to or instead of (nearly one in five Americans) their prescription medications. At the same time, the use of CAM (**Figure 1**), including acupuncture, deep-breathing exercises, massage therapy, meditation, naturopathy, and yoga, is growing, specifically among patients with chronic conditions and those taking prescription drugs (Gardiner et al., 2006; Nahin et al., 2009). As regards veterans, a longing for a holistic approach to health care and the lack of trust in the health system are more common in those who use CAM (Kroesen et al., 2002). In particular, it has been shown that 75% of veterans, as well as the general population, used vitamins and supplements, whereas 18% substituted drugs (US Food and Drug Administration, 2008). Among the latter, 25% replaced hyperlipidemia medications, 17% the anxiolytics/antidepressants, 15% those for both arthritis/back pain and hot flashes, 10% the antidiabetic drugs, and 8% those for hypertension (Goldstein et al., 2014). Patti et al. (2017) suggested that the use of nutraceuticals containing omega-3, polyphenols, vitamins, and trace elements could be useful in contrasting metabolic syndromes. In addition to this, a recent meta-analysis has pointed out the improvement of moderate hypercholesterolemia determined by supplementation with red yeast rice (Fogacci et al., 2019). It has been reported that red yeast rice supplementation is safe and not associated with increased incidence of muscular adverse effects (Fogacci et al., 2019). Furthermore, bergamot, red yeast rice, soluble fiber, berberine, artichokes, plant sterols, and stanols have been suggested as an alternative or additional therapy to statins, alone or in combination with each other (e.g., with drugs, such as ezetimibe), in statin-intolerant patients (Cicero et al., 2017; Banach et al., 2018). On the contrary, Ward et al. (2018) reported that in their clinical practice experience, 1/18 patients with statin-associated muscle symptoms (SAMS) (5.5%) had side effects after nutraceutical treatment (muscle ache/stiffness and intolerance).

On the other hand, the risk of statin-induced serious muscle injury, including rhabdomyolysis, is <0.1%, and the risk of serious hepatotoxicity is ≈0.001% (Newman et al., 2019), and in a meta-analysis, statins did not seem to modify rhabdomyolysis, myalgia, or rise in creatine kinase (Tramacere et al., 2019). It must be borne in mind that fermented red rice contains monacolin K, having the same formula of lovastatin (US National Library of Medicine^1^). In 2014, Italy was ranked first in Europe for consumption of nutraceuticals, and the presence of some problems related to their use has been reported by the Società Italiana per lo Studio dell’Aterosclerosi (SISA) (Averna and Pirro, 2017). The improper vigilance and the strong belief in the safety of natural products are cultural limits to be demolished through scientific information (Averna and Pirro, 2017). A significant percentage of people who consume nutraceuticals declared to have not purchased them as a result of medical advice (Averna and Pirro, 2017). In spite of the uncertainties about the efficacy of herbal preparations and dietary supplements, users who want to check their health personally often believe that herbal preparations and dietary supplements are natural and have fewer side effects (Wu et al., 2014). Actually, recent US data indicated that the use of a combination of dietary supplement products is most commonly associated with side effects (Austin et al., 2016; Knapik et al., 2016); furthermore, potential interactions can also occur between drugs and herbal/nutritional supplements (**Figure 1**) (Loya et al., 2009) with significant consequences, such as an increased risk of adverse drug reactions probably due to the induction or inhibition of cytochrome P450 isoenzymes (Henderson et al., 2002); for example, *Hypericum perforatum*, known for the antidepressant and sedative activity of its phytocomplex, has the ability to accelerate cytochrome P450, giving multiple interactions with different classes of drugs such as selective serotonin reuptake and monoamine oxidase inhibitor (Lantz et al., 1999), warfarin (Jiang et al., 2004), digoxin (Muller et al., 2004), statins (Sugimoto et al., 2001), and all cytochrome P450 metabolized agents (Markowitz et al., 2003). Moreover, coadministration of ephedra (*Ephedra sinica*), which can increase blood pressure and decrease platelet aggregation, and nonsteroidal anti-inflammatory drugs may potentiate the risk of cerebral hemorrhage and gastrointestinal ulcer bleeding (Meng and Liu, 2014). Possible interactions with drugs have also been suggested for mineral-fortified foods and fruit juices, which are able to influence the bioequivalence of levofloxacin and ciprofloxacin (Neuhofel et al., 2002; Amsden et al., 2003; Wallace et al., 2003). Accordingly, the Department of Veterans Affairs has dedicated a special section on its website to all the possible interactions between food and drugs, also indicating nutraceuticals (**Figure 1**) to be taken carefully if you are undergoing a polypharmacy (US Department of Veterans Affairs^2^). On one hand, drug–drug interactions are included in most pharmacovigilance systems owing to their widely recognized clinical relevance; on the other hand, nutrient–drug interactions are still underexplored, and their appraisal is not part of the clinical routine, despite being supported by a lot of data (Péter et al., 2017). Therefore, it would seem necessary to proceed with a systematic evaluation of such interactions by means of an appropriate analysis both of a possible influence of the nutritional status in the drug action and of the effect of adverse drug reactions on the nutritional status (Péter et al., 2017). This systematic analysis obviously has to provide for a nutritional appraisal throughout the phases of drug development and post-marketing surveillance. Adverse drug effects in clinical practice, in particular those related to nutrition, should be reported spontaneously, and a special attention should be paid to taking into account malnutrition, the global nutritional status, and the dietary supplements used (Péter et al., 2017). Particularly, regarding nutritional status, Becerra et al. (2016) have pointed out the extreme necessity to carry out health promotion measures among veterans in order to encourage a healthy diet in this population, especially those with limited access to healthy food options. In fact, negative dietary practices have come to light among veterans, which are associated with food insecurity. In this regard, the Department of Veteran Affairs provides guidance to veterans about how a healthy diet, rich in fruits and vegetables, accompanied by movement, can be useful in combating overweight and related diseases (Rutledge et al., 2017). Facilitating healthy diets, physical activity, and weight management in the veteran population is an important public health challenge (**Figure 1**). In fact, a cross-sectional analysis reported that approximately 37% and 33% of women and men veterans are obese, respectively (Das et al., 2005), while others demonstrated higher prevalence of overweight status (Koepsell et al., 2009) and greater waist circumference among veterans (Koepsell et al., 2012) as compared with the civilian population. Such a prevalence of overweight and obesity among veterans may be due to their dietary practices. In fact, recent studies have found that military service impacts soldiers’ food environment and food security, which then influences eating behavior and food choices both during military service and following discharge (Smith et al., 2009; Wang et al., 2015; Widome et al., 2015). During this time frame, veterans consume high-fat and high-carbohydrate foodstuffs, with a preference for specific food items (burgers and fries) (Smith et al., 2009), which is influenced by their low cost, an important aspect that could further be driving vulnerable populations away from healthier items, which usually are more expensive (Drewnowski and Darmon, 2005a; Drewnowski and Darmon, 2005b; Jetter and Cassady, 2006).

A diet high in fruits and vegetables is associated with decreased risk for chronic diseases such as cardiovascular disease, hypertension, diabetes, and cancer (Adams and Standridge, 2006); therefore, it may play an important role in reducing veterans’ health risks.

In our opinion, the Mediterranean Diet Pyramid could be the basis for integrative medicine for veterans with disabilities, but patient-centered and interprofessional approaches represent the real added value for the best health care management. A comprehensive approach should also include physical medicine and rehabilitation clinicians, pharmacists, and nutritionist in order to prevent malnutrition, self-prescription of CAM, and food–drug and/or nutraceutical–drug interactions according to a biopsychosocial model (**Figure 1**) (Ciccotti et al., 2018). Personalized health care for chronic noncommunicable diseases that impact quality of life should consider gut microbiota, genetic and epigenetic factors (Peluso et al., 2018a), and moods and hormones involved in stress response (Peluso et al., 2018b) rather than functional status. In particular, as previously suggested, in order to avoid potential food–drug interactions, plant foods should be chosen within those containing low phytochemicals and high micronutrients. If this aim is difficult to reach, vitamin and/or mineral supplementation can be recommended (top of the pyramid for veterans, Ciccotti et al., 2018).

## Author Contributions

MC, PA, and PM developed the concept and wrote the paper. DM contributed to the bibliographic research. TS coordinated the research and conception work. MP, FL, CB, and IP drafted the work and revised it for important intellectual content.

## Funding

This work is an activity of the project AMAMP (2019-2021), funded by Ministero della Difesa, Italy.

## Conflict of Interest Statement

The authors declare that the research was conducted in the absence of any commercial or financial relationships that could be construed as a potential conflict of interest.

## Footnotes

1 US National Library of Medicine. Available online at https://pubchem.ncbi.nlm.nih.gov/#query=Monacolin%20K

2 US Department of Veterans Affairs. Available online at https://www.nutrition.va.gov/Food_Drug_Interactions.asp (last access 06-11-2018).
